# 40 Hz Light Flicker Alters Human Brain Electroencephalography Microstates and Complexity Implicated in Brain Diseases

**DOI:** 10.3389/fnins.2021.777183

**Published:** 2021-12-13

**Authors:** Yiqi Zhang, Zhenyu Zhang, Lei Luo, Huaiyu Tong, Fei Chen, Sheng-Tao Hou

**Affiliations:** ^1^Brain Research Centre and Department of Biology, Southern University of Science and Technology, Shenzhen, China; ^2^Department of Neurosurgery, The First Medical Center, Chinese PLA General Hospital, Beijing, China; ^3^Department of Electrical and Electronic Engineering, Southern University of Science and Technology, Shenzhen, China

**Keywords:** EEG, microstate, LZC complexity, 40 Hz flicker, AD, stroke, therapeutics

## Abstract

Previous studies showed that entrainment of light flicker at low gamma frequencies provided neuroprotection in mouse models of Alzheimer’s disease (AD) and stroke. The current study was set to explore the feasibility of using 40 Hz light flicker for human brain stimulation for future development as a tool for brain disease treatment. The effect of 40 Hz low gamma frequency light on a cohort of healthy human brains was examined using 64 channel electroencephalography (EEG), followed by microstate analyses. A random frequency light flicker was used as a negative control treatment. Light flicker at 40 Hz significantly increased the corresponding band power in the O1, Oz, and O3 electrodes covering the occipital areas of both sides of the brain, indicating potent entrainment with 40 Hz light flicker in the visual cortex area. Importantly, the 40 Hz light flicker significantly altered microstate coverage, transition duration, and the Lempel-Ziv complexity (LZC) compared to the rest state. Microstate metrics are known to change in the brains of Alzheimer’s disease, schizophrenia, and stroke patients. The current study laid the foundation for the future development of 40 Hz light flicker as therapeutics for brain diseases.

## Introduction

In mouse models of Alzheimer’s disease (AD) and stroke, low gamma frequency light flickers at frequencies from 30to 50 Hz entrain brain oscillations in the visual cortex and hippocampus. Such entrainment has neuroprotective effects in both models of AD and stroke (Iaccarino et al., 2016; Etter et al., 2019; Zheng et al., 2020). The neuroprotective mechanism conferred by low gamma light flicker treatment is different in AD and stroke. Activation of microglia reducing brain Aβ load is considered as the primary mechanism in light flicker protected AD mouse brain (Iaccarino et al., 2016; Adaikkan et al., 2019) while enhancing presynaptic excitatory neurotransmission is the primary mechanism of neuroprotection in a mouse model of stroke as we have shown (Zheng et al., 2020). Nevertheless, more evidence is emerging showing that light flicker entrainment in the brain affects a wide range of physiological processes, including circadian rhythms and sleep (Yao et al., 2020a,b). As such, the feasibility of using 40 Hz light flicker to treat human neurological diseases is actively explored and debated (Duecker et al., 2021; He et al., 2021). The main aim of the present study was to determine the effect of 40 Hz light flicker visual stimulation in healthy human brains using multichannel (64 channels) electroencephalography (EEG). By comparing with reported changes in diseased human brain EEG, our short-term aim is to determine the effect of 40 Hz light flicker on normal healthy human brain EEG. The long-term goal is to establish whether a 40 Hz light flicker may have a therapeutic effect.

Electroencephalography is a low-cost, non-invasive measure of neuronal electrical activity in the brain. EEG is widely used in healthcare systems around the world for the diagnosis of neurological diseases such as AD, stroke, and epilepsy (Smith, 2005). EEG microstates analysis is emerging as a powerful tool for studying the temporal dynamics of whole-brain neuronal networks, which has been argued to have the potential to serve as a non-invasive functional biomarker of neurological diseases (Nishida et al., 2013; Milz et al., 2017; Michel and Koenig, 2018; van der Zande et al., 2018; Musaeus et al., 2019; Tait et al., 2019, 2020; da Cruz et al., 2020; Klepl et al., 2021). Microstates describe a small number of topographic classes which are stable for periods of tens or hundreds of milliseconds before rapidly transitioning to another class (Dierks et al., 1997; Lehmann et al., 1998; Koenig et al., 2002; Smailovic et al., 2019; Tait et al., 2020). Microstate rapid transitioning is believed to represent the electrophysiological correlates of the quick activation and inactivation of the brain’s resting-state networks relating to different functions (Koenig et al., 2002; Michel and Koenig, 2018; Musaeus et al., 2019; Tait et al., 2020). This is why EEG microstates are nicknamed “atoms of thought” (Lehmann et al., 1998). Demonstration of EEG microstates coverage, transitioning, and complexity changes in response to light flicker treatment, therefore, might reveal biomarkers for exploring the changes in brain information processing and cognitive function dynamics on the millisecond scale. Modulation of microstate changes may also represent a potential method to improve the functionality of diseased brains.

In the present study, we used EEG to determine brain responses to 40 Hz light flicker in a cohort of 20 healthy young adults. Data demonstrated that 40 Hz light flicker significantly increased the overall brain oscillation power, particularly in the occipital areas on both sides of the brain. Importantly, compared with the rest state, the 40 Hz light flicker significantly altered the coverage, transitioning duration, the Lempel-Ziv complexity (LZC), all of which are typically altered in diseased patients due to regional inactivation. These data laid the foundation for further studies to establish 40 Hz light flicker as therapeutics for neurological diseases such as AD, schizophrenia, and stroke.

## Materials and Methods

### Participants

Data from 20 healthy individuals (*n* = 16 males, *n* = 4 females, aged 20 years old) who have not suffered from any neurological disease in the past 5 years were collected. All participants were free from medication known to affect cognition. All participants provided written informed consent before participating and were free to withdraw at any time. All procedures were carried out in accordance with the relevant guidelines and regulations. Procedures were in accordance with the Declaration of Helsinki and approved by local ethics committees, i.e., the Southern University of Science and Technology Institutional Review Board (SUSTech IRB 2021087). Data from all subjects were included, and the subjects were identified by aliases from S1 to S20.

### Visual Stimulation Protocol

The visual stimulation equipment consisted of two modules, a programmed tunable frequency signal generator and a LED lighting unit. The LED lighting unit consisted of six LED strips evenly fitted on a 30 cm × 20 cm plane board. Each LED strip was 50 cm long (12 V) fitted with 18 individual LED light bulbs. The specific frequency of light flickers was generated using the signal generator. The driving signal (0–5 V square wave, 0.1–120 Hz, even distribution) was generated by an Arduino UNO and amplified by a pulse-width modulation signal amplifier. The input voltage signal for LED strips alternated between 12 and 0 V, and the corresponding luminance switched between maximum LED luminance and complete dark. Dynamic luminance curve was verified using photosensitive diodes and oscilloscope, confirming a stable frequency and square wave-shaped illuminance. Two types of flicker signals, i.e., 40 Hz and random frequency, were produced as described previously (Zheng et al., 2020). The 40 Hz signal has a consecutive 2.5 ms cycle. A random flicker luminance was produced as lights were on and off for a randomized interval > 0 that averaged 12.5 ms. For example, random stimulation with off intervals was ranged from 0.2 to 34.1 ms. Therefore, the frequency of light flickering was random. The luminance switches between maximum LED luminance and complete dark. Eyes-opened, 30 s of resting-state EEG was collected from all subjects. The same test was also performed on subjects with one side-eye covered to allow only one eye exposure to light flicker.

All subjects sat at a comfortable chair and looked at a fixation point on a computer screen 40 cm away, which continuously displayed light flickers at the specified frequencies for 30 s. The participant was allowed to rest for 30 s and followed by another 30 s of light flicker while the EEG was collected.

### Electroencephalography Data Collection and Pre-processing

Scalp EEG signals were acquired using a 64 channels Quick-Cap (Compumedics Neuroscan according to the standard 10/20 System) with a Neuroscan SynAmps2 amplifier (Neuroscan, Inc.). All electrodes were referenced to CPz and grounded at AFz according to the manufacturer’s recommendation. The whole experiment was conducted in a double-walled electrically and acoustically shielded room.

Before the experiment, the electrode impedances were adjusted under 20 kΩ, and the left and right mastoid reference (M1 and M2) were used for EEG measurement. The sampling frequency was 500 Hz. The pre-processing of the EEG data was done using the MNE-Python package (V0.23.0) (Gramfort et al., 2013). A band-pass filter range of 0.1–100 Hz and a 50 Hz notch filter was applied during the pre-processing. The Common Average Reference (CAR) was used, which eliminated artifacts using the average of all electrodes. CAR was calculated by subtracting the average value of data of all electrodes from the data of the selected channel, and its formula was shown in Eq.1, where *N* represents the total number of electrodes, $V_{i}^{CAR}$ is the data of *i*th electrode after CAR processing, $V_{i}^{RAW}$ is the original data of *i*th electrode.


(1) $$V_{i}^{CAR}=V_{i}^{RAW}-\frac{1}{N}\Sigma_{j=1}^{N}V_{j}^{RAW}$$

The independent components analysis (ICA) function in the MNE-Python package was used to remove the artifacts automatically. The ocular and muscular artifacts were removed using the ICA method. Briefly, the visual inspection of epochs was performed by a trained EEG researcher to identify the presence of a minimum of artifacts, such as muscle activity, eye blinks, and drowsiness. The epochs were replaced by other epochs or the EEG was excluded from analyses when artifact epochs were identified.

### Extract the Relative Power of Electroencephalography Signals

To calculate EEG signals’ relative power (percentage power), we first extracted a specific frequency band (39–41 Hz) from all EEG signals. The absolute power of a specific frequency band was calculated by integrating the power spectral density (PSD) of that frequency range. The *mne.time_frequency.psd_multitaper()* in MNE-Python package was used to calculate the PSD (Slepian, 1978; Percival and Walden, 1993).

The absolute power of a specific position is the average power calculated from several adjacent electrodes. Then the relative power of one frequency band is the percentage of the absolute power of the frequency band relative to the total frequency band power, and its formula is shown in Eq.2 and Eq.3, where the *T* represents the total frequency band power, *a*_*f*_ represents the absolute power of frequency band *f*, *r*_*f*_ represents the relative power of frequency band *f*:


(2) $$T=\Sigma_{f}a_{f}$$

(3) $$r_{f}=\frac{a_{f}}{T}$$

### Decibel Unit

The power or intensity ratio can be expressed as decibels by calculating the ratio of the measured value to the reference value based on the logarithm of 10 and multiplying it by 10. When displaying the power spectrum density (PSD), we showed the PSD map in dB unit, and its calculation formula is as follows, where *L*_*dB*_ means the value in *dB* unit, and *L*_*o*_ represents the original value:


(4) $$L_{dB}=10{\text{log}}_{10}L_{o}$$

### Microstate Extraction

Microstates extraction and analyses were performed exactly as described previously (Musaeus et al., 2019, 2020) to facilitate comparisons. To reduce noise, we rejected microstate segments shorter than 30 ms. This was to ensure that the short segments, which may have been due to noise, did not affect the results. The clean, pre-processed epochs of each condition are concatenated as single long epochs. The spatial standard deviation of the EEG topography was calculated at each time point across all electrodes as global field power (GFP). The GFP is an instant and reference-independent measure of the whole-brain neuronal activity (Lehmann and Skrandies, 1980). For each subject in a different condition, the GFP was calculated separately. Only the relatively stable EEG topographies around these GFP peaks were further analyzed (Koenig et al., 2002). Briefly, EEG alpha band (8–13 Hz) were filtered, and the maps at the GFP peaks were extracted for a modified *k*-means spatial cluster analysis. This way, the mean microstate topographies for each class were produced. The output contains clustered microstate maps, global explained variance, and a time course containing the microstate at each GFP peak. Because the polarity of microstates is ignored in the definition of microstates, only the spatial configuration of a dipole was collected. The modified *k*-means clustering was performed to identify four common classes of microstates (Nishida et al., 2013; Michel and Koenig, 2018; Musaeus et al., 2019, 2020; Tait et al., 2020). Four microstates from the rest states, random flicker and 40 Hz light flicker-treated groups, were identified through back-fitting the global maps to each of the EEG files by labeling each of the EEG segments with the class of microstates.

Additional measures were implemented to make sure the specificity of microstate classes. A common average reference (CAR) method was used to increase the signal-to-noise ratio by enhancing the control signal and/or reducing noise. The average value of the entire electrode montage (the common average) was subtracted from the channel of interest (McFarland et al., 1997; Ludwig et al., 2009). The independent components analysis (ICA) was also used to remove ocular and muscular artifacts (Sun et al., 2005). In addition, because the polarity was ignored in the definition of microstates, together, these procedures established specific microstate classes.

### Microstate Analysis

By back-fitting the clustered microstates map to the original EEG data, we calculated the global explained variance (GEV), duration, coverage and performed the Markov chain analysis. The term GEV refers to the variance of the EEG data that can be explained by the clustered microstate maps. The microstate coverage represents the fraction of total recording time that the microstate is dominant (Lehmann et al., 1987). The term duration is the average time of a particular microstate map to remain stable without transitioning to the next state.

To identify the transition probability of different classes of microstates, we performed the Markov chain analysis, which can be used to describe the dynamics of a system with different states (Baudoin, 2010). Markov chain represents the probability distribution for transitioning to a different state or keeping the current state at the next time point. In this study, the sampling frequency of EEG recording was 500 Hz; therefore, the time resolution of the Markov chain analysis was 2 ms. Due to the existence of self-transitioning (the next state is the same as the previous state), two separate Markov chains were computed for different conditions (rest state and 40 Hz frequency flicker). The Markov chain with self-transitioning-allowed was used to test the stability of microstates, and the Markov chain without self-transitioning was used to test the transition pattern when microstate transitions did occur.

### Microstate Lempel–Ziv Complexity Analysis

The Lempel–Ziv complexity (LZC) of the microstate transitioning sequence was calculated as previously described (Tait et al., 2020).

To ensure comparability, we employed 1–200 Hz filter for EEG files as described by Tait et al. (2020). We computed the LZC of each microstate time course, in which microstate at different time points was denoted in integers from 0 to 3. As determined by the algorithm, length has an inevitable impact on the value of LZC. Therefore, epoch lengths were equalized to identical lengths prior to computation.

### Cortical Source Estimation

Electroencephalography data were subjected to source estimation to determine cortical initiators of observed potential on sensors, that is, to map the current activity on the brain cortex. We computed a dynamic statistical parameter map (dSPM) for source estimation, which normalizes the estimated current amplitudes at each location to their respective standard error of the estimate. A prerequisite for computing dSPM is a noise covariance matrix. Using the resting-state EEG recording of the 30 to 20 s before every epoch of flicker stimulation, a noise covariance matrix was computed. The forward solution, defining the transformation projecting electrode positions into Cartesian coordinates with Right-Anterior-Superior orientation, and subsequent analysis are computed based on the fsaverage surface template (Desikan et al., 2006). Note that different from the traditional approach that explicitly computes an inverse operator for dSPM, the implementation we employed computes a singular value decomposition (SVD) of a matrix composed of the noise-covariance matrix, the result of the forward calculation, and the source covariance matrix. This approach has the benefit that the regularization parameter (signal to noise ratio) can be adjusted easily when the final source estimates or dSPMs are computed as reported previously (Dale et al., 2000).

### Statistics

All data were analyzed using customized scripts developed in Python 3 (v.3.8.0), R (v.4.0.3), and GraphPad Prism software (v.8.0.2). To determine differences between different states (rest state, 40 Hz light flicker stimulation, random light flicker stimulation), we performed a one-way ANOVA for the three groups (if both session datasets were normally distributed), and the normality was checked using the One-sample Kolmogorov–Smirnov test and Shapiro–Wilk normality test. For multiple comparisons in the analysis of percent power, microstate coverage, duration, occurrence and LZC, we performed FDR correction using the two-stage linear step-up procedure of Benjamini, Krieger, and Yekutieli (GraphPad Prism V8) for all *P* values to reduce the risk of type I error. The *post hoc* tests were considered significant with an adjusted *P* < 0.05. For multiple comparisons in the analysis of transition, One-way ANOVA with Tukey’s *post hoc* test was used to compare the transition probability in different conditions.

## Results

### Increase Brain Electroencephalography Power Associated With 40 Hz Light Flicker Stimulation

**FIGURE 1:**
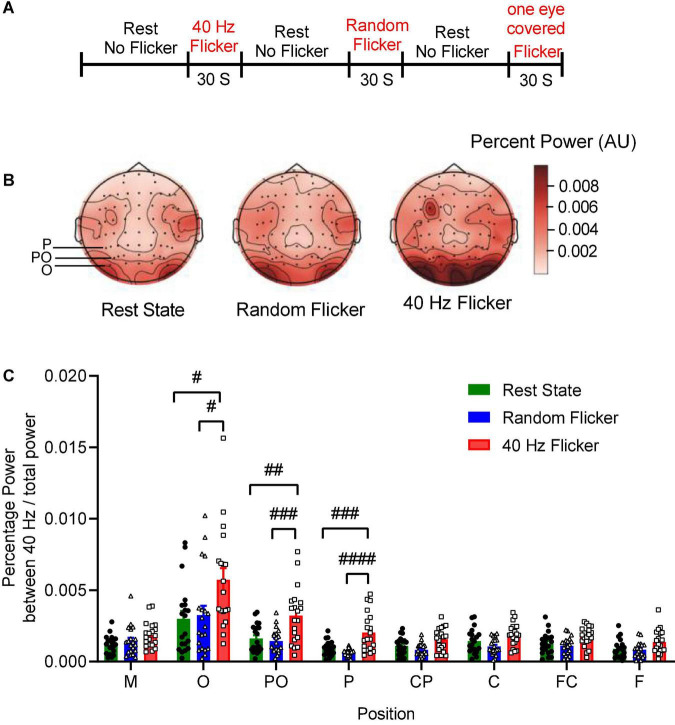
EEG sequence and PSD analysis. Panel **(A)** depicts the experimental schema of light flicker stimulation and EEG sequence. Panel **(B)** is a graphical representation of the intensities of EEG signals in the anatomical brain regions, illustrating the topographic maps of PSD change (Left: rest state; Middle: random flicker; right: 40 Hz light stimulation). Selected positions of O, PO, and P were indicated on the rest state map. The scale bar represents an arbitrary unit (A.U.). EEG recordings were analyzed, and the percentage of the power spectrum between 40 Hz and the total power was calculated from different brain regions as indicated in panels **(C)**. Statistical comparisons between the three conditions (rest state, random flicker, and the 40 Hz light stimulation) were performed using one-way ANOVA with multiple comparisons using FDR correction using the two-stage linear step-up procedure of Benjamini, Krieger, and Yekutieli (GraphPad Prism v8). Significant groups were shown in panel **(C)**. #, ##, ###, and #### indicate the FDR adjusted *P* < 0.05, *P* < 0.01, *P* < 0.001, and *P* < 0.0001, respectively.

Test participants consisted of 16 males and four females. The participants were randomly chosen within the same age group at 20 years old. EEG was performed in a quiet and electrical shielded room using the event sequence shown in Figure 1A. All test data were included in the final analyses. The EEG PSD data from three subjects (S14, S17, and S18) were excluded as shown in Figure 1C and Supplementary Figure 1, because they were identified as outliers by the box plot with the 1.5 × IQR rule. This anomaly may be due to technical issues during the data recording process.

To investigate the relationship between 40 Hz light stimulation and EEG power changes, we analyzed the power spectrum changes in three situations: rest state (open eye rest and without light flicker), 40 Hz light flicker stimulation, and random frequency light flicker stimulation. Graphical representation of the intensities of EEG signals percent power changes in the anatomical brain regions in the form of topography maps were shown in Figure 1B, illustrating the noticeable enhancement of EEG signals in the occipital areas corresponding to the O, PO, and P areas covered by the electrodes.

We artificially separated the brain into seven different areas based on the electrode positions along the axis from the frontal lobe to the occipital lobe, i.e., F, FC, C, CP, P, PO, O. The position M represented the reference electrode M1 and M2. The percent powers as 40 Hz / total power (0.1–100 Hz) at the seven brain regions were calculated, and the comparisons amongst the three groups were shown in Figure 1C. The *post hoc* test showed 40 Hz light flicker stimulation significantly increased the percent power of brain regions at O, PO, P, CP, FC, and F (Figure 1C). Specifically, in position O, a significant increase of percent power occurred between the rest state and 40 Hz light stimulation (False Discovery Rate (FDR) adjusted *P* = 0.0064, *t*-value = 2.848, df = 56), and between random flicker and 40 Hz light stimulation (FDR adjusted *P* = 0.0065, *t*-value = 2.584, df = 56) (Figure 1C and Supplementary Figure 1). There was no significant difference in EEG percentage power between rest and random flicker. Similarly, in position PO, significant differences were found between rest state and 40 Hz light stimulation (FDR adjusted *P* = 0.0002 *t*-value = 3.718, df = 57), and between random flicker and 40 Hz light stimulation (FDR adjusted *P* = 0.0001, *t*-value = 4.153, df = 57). Furthermore, significant differences were also found in position P, with a significant difference between rest state and 40 Hz light stimulation (FDR adjusted *P* < 0.0001, *t*-value = 4.024, df = 57), and between random flicker and 40 Hz light stimulation (FDR adjusted *P* < 0.0001, *t*-value = 5.192, df = 57). In contrast, random frequency light flicker stimulation did not have such an effect when compared with the rest state (FDR adjusted *P* > 0.1) (Figure 1B and Supplementary Figure 1A).

### 40 Hz Light Flicker Entrainment

**FIGURE 2:**
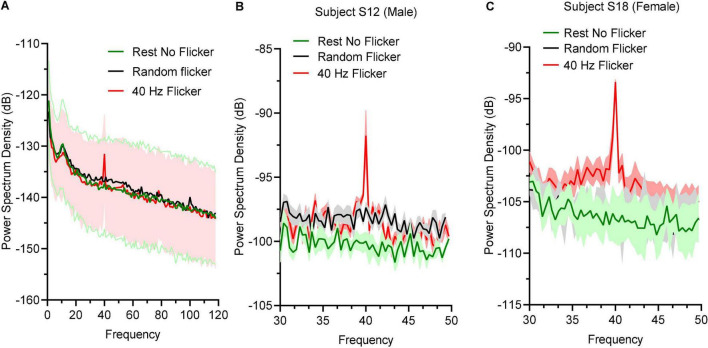
Power spectral density responses during rest state and light flicker stimulation. **(A)** Quantification of PSD in different states displayed in decibel (dB) unit. Thick lines in green, black, and red showed the mean values and shaded areas represented SEM; **(B)** PSD between 30 and 50 Hz of a male subject **(B)** and a female subject **(C)**.

To determine the effect of 40 Hz light flicker to the human brains, power spectral density (PSD) at O electrodes was calculated. Oscillation entrainment occurred in response to 40 Hz light flickers, but not the rest state or arrhythmic random frequency light flicker (Figure 2A). The PSD of a male subject S12 (Figure 2B) and a female subject S18 (Figure 2C) were also shown to demonstrate a similar level of entrainment of 40 Hz light flicker in both sexes.

### Identification of 40 Hz Light Flicker Stimulation-Induced Topological Microstates

**FIGURE 3:**
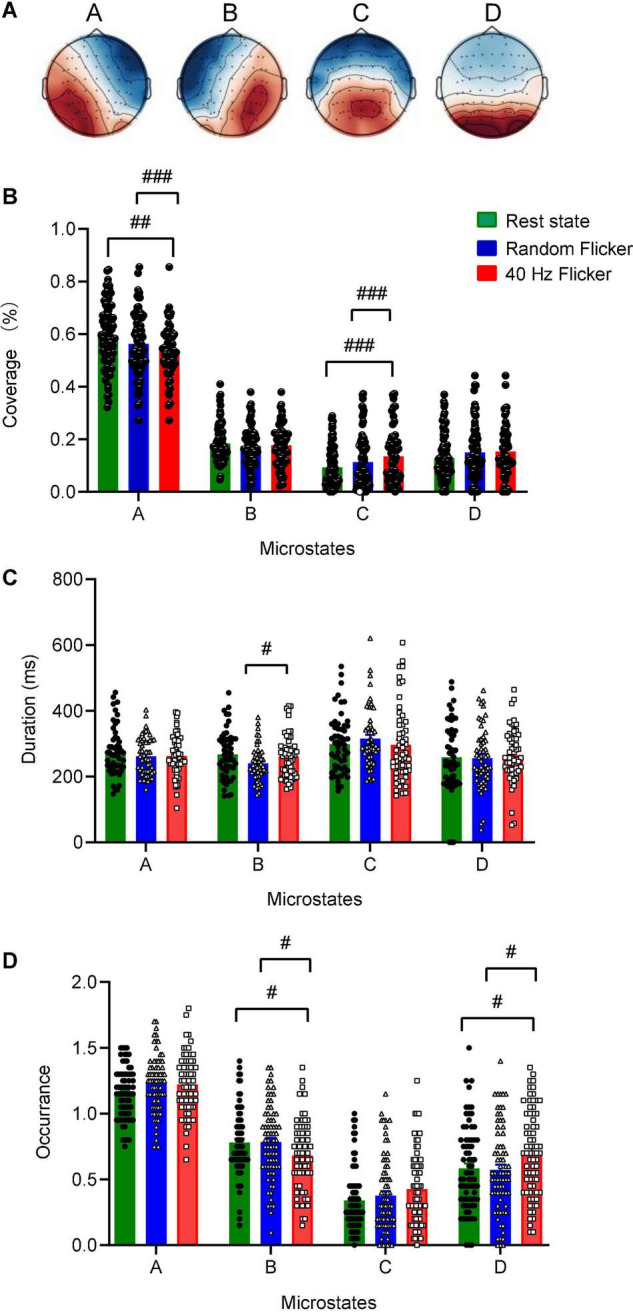
Identification of microstate classes, coverage, duration, occurrence. Panel **(A)** The global maps were calculated based the aggregated dataset from all participants and were back-fitted to each of the EEG recordings. The labels **(A–D)** indicate the four microstate classes. The four microstate classes coverage **(B)**, duration **(C)**, and occurrence **(D)** were calculated and shown. Statistical comparisons between the three conditions (rest state, random flicker, and the 40 Hz light stimulation) were performed using one-way ANOVA with multiple comparisons using FDR correction using the two-stage linear step-up procedure of Benjamini, Krieger, and Yekutieli using GraphPad. Significant groups were shown in panel **(B–D)**. #, ##, and ### indicate the FDR adjusted *P* < 0.05, *P* < 0.01, and *P* < 0.001, respectively. Error bars are S.E.M.

The global maps calculated based on the aggregated dataset from all participants were back-fitted to each of the EEG recordings to generate typical clustered four topographies. Four microstates with fixed polarities were identified using the modified K-means clustering method, as shown in Figure 3A. In the rest state, the four microstate classes largely align with the classical microstate classes A-D associated with wakeful rest (Lehmann et al., 1998; Nishida et al., 2013; Milz et al., 2017; Michel and Koenig, 2018; Musaeus et al., 2019; Tait et al., 2020). These four classes of microstates are electrophysiological correlates of the auditory (A), visual (B), saliency (C), and frontoparietal working memory/attention (D) of the resting-state networks (Michel and Koenig, 2018).

Microstate coverage was calculated and compared across treatment groups (Figure 3B). 40 Hz light flicker stimulation significantly reduced the coverage of microstate class A (FDR adjusted *P* = 0.0047, *t*-value = 2.643, df = 174), but increased microstate class C (FDR adjusted *P* = 0.0010, *t*-value = 3.233, df = 174) in the brain (Figure 3B). It was noted that 40 Hz light flicker also showed significantly reduced coverage microstate classes A (FDR adjusted *P* = 0.0001, *t*-value = 3.817, df = 174) and C (FDR adjusted *P* = 0.0010, *t*-value = 3.162, df = 174) compared with random flicker treatment.

### Altered Microstate Duration, Occurrence, and Markovian Syntax Transition Matrix During 40 Hz Light Flicker Stimulation

We next compared microstate duration, occurrence and transition sequence behaviors during rest state and in response to random flicker and 40 Hz light flicker stimulation. The mean duration of all microstates in all three conditions was similar except that 40 Hz light flicker increased microstate class B duration compared with the random flicker treatment (FDR adjusted *P* = 0.0300, *t*-value = 2.482, df = 135) (Figure 3C). Interestingly, the occurrence of microstate B and D significantly increased in 40 Hz flicker group compared with the rest state (For microstate B: FDR adjusted *P* = 0.0141, *t*-value = 2.229, df = 216; For microstate D: FDR adjusted *P* = 0.0141, *t*-value = 2.338, df = 216) and random flicker treatment (For microstate B: FDR adjusted *P* = 0.0158, *t*-value = 2.184, df = 216; For microstate D: FDR adjusted *P* = 0.0158, *t*-value = 2.400, df = 216) (Figure 3D).

**FIGURE 4:**
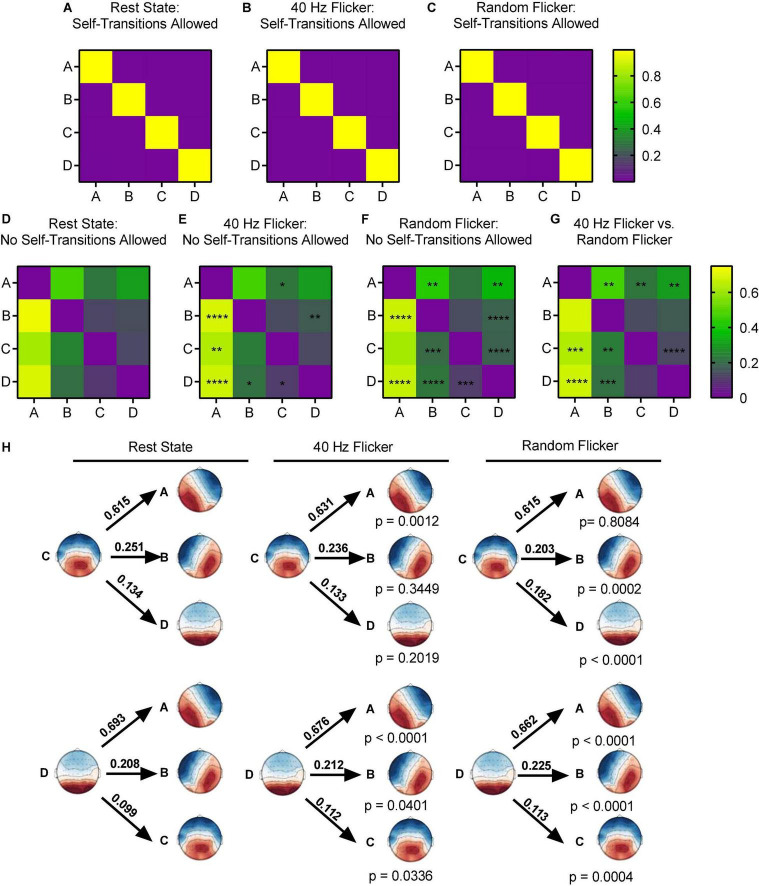
Microstate transition syntax analysis in different conditions. Panels **(A–G),** each map displays Markov chain analysis result for one of the four microstate classes. **(A–C)** Self-transition allowed tests, and **(D–G)** for self-transition not allowed tests. Panel **(H)** shows a schematic view of the syntax pattern to indicate transition from C and D to other states in the rest state (left), 40 Hz flicker (middle), and random flicker (right). A one-way ANOVA with Tukey *post hoc* multiple comparison test was used to identify significant groups. *, **, ***, and **** indicated the Tukey adjusted *P* < 0.05, *P* < 0.01, *P* < 0.001, and *P* < 0.0001, respectively. Scale bars represent the probability (Prob).

Using the Markov chain analyses, we analyzed the microstate transitions syntax in different conditions. When self-transition was allowed, all microstates in the rest state, random flicker and 40 Hz light flicker states were more likely to self-transition (transition probability was about 0.9) (Figures 4A–C).

When self-transition was not allowed (Figures 4D–G), microstate class A in 40 Hz group was significantly more likely to transition to C (Tukey adjusted *P* = 0.0494, *q*-value = 3.618, df = 18) and microstate class B was significantly more likely to transition to D (Tukey adjusted *P* = 0.0043, *q*-value = 5.259, df = 18). Microstate class B was significantly less likely to transition to A (Tukey adjusted *P* < 0.0001, *q*-value = 9.323, df = 18). Microstate class C in 40 Hz flicker group was significantly more likely to transition to A (Tukey adjusted *P* = 0.0012, *t*-value = 8.110, df = 18), and class D was significantly more likely to transition to A (Tukey adjusted *P* < 0.0001, *q*-value = 9.877, df = 18) and to B and C (*P* = 0.0401 and *P* = 0.0336, respectively) (Figure 4H).

In contrast, random flicker treatment class A was significantly less likely to transition to class B but significantly more likely to transition to D (*P* = 0.0029 and *P* = 0.0024, respectively). In addition, class B was significantly less likely to transition to A and significantly more likely to transition to D (*P* < 0.0001 and *P* < 0.0001, respectively). Random flicker treatment class C was more likely to transition to B and D (*P* = 0.0002 and *P* < 0.0001, respectively), and class D more likely transition to A, B, and C (*P* < 0.0001, *P* < 0.0001, *P* = 0.0004, respectively) (Figure 4H).

### Increased Microstate Complexity During 40 Hz Light Flicker Stimulation

**FIGURE 5:**
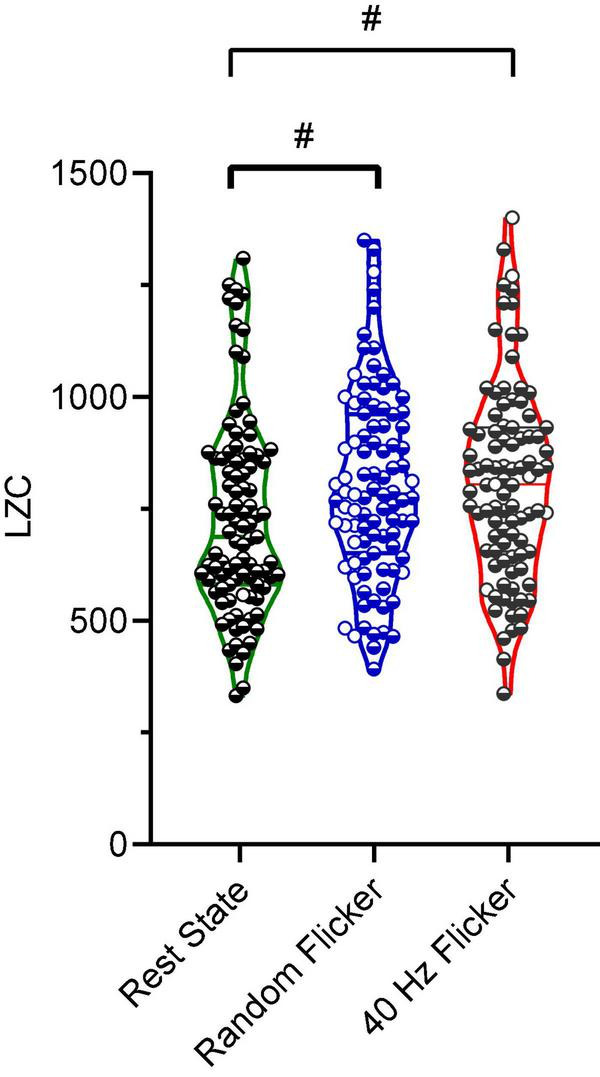
Microstate LZC. Microstate LZC under rest state, random light flicker, and 40 Hz light flicker exposure. LZC of microstate transitioning was computed for epochs with identical lengths under different conditions. Each dot referred to a single epoch during the experiment. # indicated *P* < 0.05 using one-way ANOVA with FDR *post hoc* analysis.

Studies have suggested that microstate transitioning is non-Markovian (von Wegner et al., 2017). Therefore, the Lempel-Ziv complexity (LZC) of the microstate transitioning sequence was additionally calculated. A significant increment in microstate complexity occurred in both random flicker (FDR adjusted *P* = 0.0104, *t*-value = 2.834, df = 282) and 40 Hz light flicker stimulation (FDR adjusted *P* = 0.0423, *t*-value = 2.040, df = 282) (Figure 5) compared with the rest state. Microstate LZC was regarded as an important measure of brain function impairment in AD, in which microstate complexity LZC was significantly reduced (Tait et al., 2020).

### Differential Cortical Activity Responses to 40 Hz and Random Light Flicker Stimulation

**FIGURE 6:**
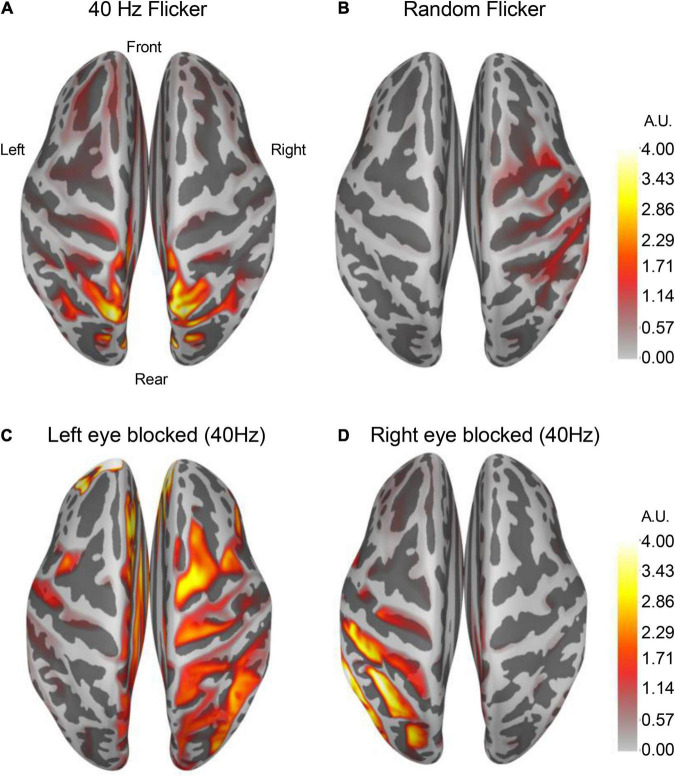
Altered cortical responses in response to 40 Hz light flicker stimulation. The brain cortical neuronal response was determined using dSPM. Average dSPMs pseudo-colored maps of brain cortical representations of increased responses to light flickers were shown in panels **(A)** (40 Hz light flicker) and **(B)** (random like flicker). The left [panel **(C)**] or the right [panel **(D)**] side of the eye was covered during the light flicker test, and the neuronal response in the brain showed a robust ipsilateral increase in response to 40 Hz light flicker stimulation (*n* = 4). The colors in the scale bar represent the mapped current density on the corresponding location in arbitrary units (A.U.).

By averaging over 100 epochs’ source estimation during light flicker stimulations, conserved cortical regions responding to 40 Hz (Figure 6A) and random light flickers (Figure 6B) were identified and mapped to the brain. In response to 40 Hz light flicker stimulation, the maximally activated region symmetrically appeared at the superior parietal cortex of both hemispheres (Figure 6A). In contrast, the random frequency light flicker induced a much lower level of activation in the cortical regions than the 40 Hz light flicker, and the areas of activation were asymmetrically distributed with a bias toward the right hemisphere (Figure 6B).

To demonstrate that cortical response to light flicker was specific, the test subject’s left eye or the right eye was blocked (covered) from light stimulation. A strong increased cortical response occurred only on the ipsilateral side of the superior parietal cortex (Figures 6C,D), confirming the observation of cortical responses to 40 Hz light flicker stimulation.

## Discussion

This study demonstrated the effects of 40 Hz low gamma frequency light flicker in healthy human brains using EEG. In responses to 40 Hz light flicker, the overall brain power increased significantly compared with the random light flicker treatment. Importantly, microstate analysis identified significantly altered microstate coverage, duration, transitioning, and LZC. Almost all of these parameters have been reported in the literature to be changed in diseased brains such as AD, schizophrenia, and stroke (Mecarelli et al., 2011; Nishida et al., 2013; Liu et al., 2016; Milz et al., 2017; Zappasodi et al., 2017; Musaeus et al., 2019, 2020; Smailovic and Jelic, 2019; Briels et al., 2020; da Cruz et al., 2020; Sebastián-Romagosa et al., 2020; Tait et al., 2020; He et al., 2021; Klepl et al., 2021).

Our test subjects have not previously been exposed to 40 Hz or arrhythmic light flickers. This experimental paradigm gives us the advantage of studying the brain responses and determining the newly formed microstate dynamics in response to 40 Hz light flickering. In order to develop this technology for clinical use, we carefully analyzed reactions in the brain of both male and female test subjects. There were no differences in entrainment responses compared between the male and female test subjects. A multi-episode of light flicker exposures was also used to determine whether there would be an altered response in entrainment. Multiple epochs from each light-flickering session were calculated and compared with data from the immediate next session. No difference in the level of entrainment to 40 Hz light flicker occurred. Moreover, this response in the brain was specific to the light treatment since it disappeared on the same side of the brain when one eye was closed/blocked during light flickering. These data demonstrated that 40 Hz light flicker consistently produced entrainment in test subjects’ brain, confirming previous animal studies (Iaccarino et al., 2016; Adaikkan et al., 2019; Zheng et al., 2020). Current studies demonstrated that short time exposure to light flicker stimulation was safe and tolerable by the test subjects. A longer-term treatment for 4 to 8 weeks at 1 h per day was also reported to be safe and tolerable by human test subjects (He et al., 2021).

Electroencephalography microstate analysis parses EEG signals into topographies representing discrete, sequential network responses. Methodologies used in the literature to generate microstates are varied, and several considerations are taken into account when designing microstate analysis. Given that higher frequencies are especially prone to be contaminated by muscle artifacts, alpha band-pass (8–13 Hz)-filtered EEG is commonly used in the microstate literature (Milz et al., 2017; von Wegner et al., 2017; Musaeus et al., 2019). More importantly, strategies for extracting and analyzing microstates are important. As described in the studies by Musaeus et al. (2019, 2020), the microstates were obtained from the aggregated dataset from all participants and then back-fitted to the data of each condition. Thus, differences between healthy and diseased brains (e.g., duration, the difference in transition probabilities, etc.) are described in terms of the same set of canonical microstates. This method is in stark contrast to the one used by Tait et al. (2020) in that the band-filtered EEG files from each condition were extracted and analyzed.

To facilitate comparisons with the most described diseased brain microstates in the literature, our microstates extraction and analyses were performed exactly as described by Nishida et al. (2013), Milz et al. (2017) and Musaeus et al. (2019) using the alpha band-pass-filtered EEG. To reduce noise, we rejected microstate segments shorter than 30 ms to ensure that the short segments, which may have been due to noise, did not affect the results. The clean, pre-processed epochs of each condition were concatenated as single long epochs. One of the main criteria we used to determine each microstate class was that it must have a dominant presence among all the microstates in order to be considered as an independent class. This way, the resting state microstate classes obtained in the current study aligned well with those reported in these papers in terms of the four microstate coverage, duration, and occurrence.

Previous studies have shown that AD patients exhibited a pattern of global microstate disorganization associated with the frontoparietal working-memory/attention network inactivation. Alteration of microstate metrics has become a promising indicator to demonstrate brain network disruptions in mild cognitive impairment (MCI), AD, schizophrenia, stroke, epilepsy, and dementia (Mecarelli et al., 2011; Finnigan and van Putten, 2013; Nishida et al., 2013; Michel and Koenig, 2018; van der Zande et al., 2018; Musaeus et al., 2019, 2020; Smailovic and Jelic, 2019; da Cruz et al., 2020). Compared with these studies, 40 Hz light flicker tended to show an opposite effect to those described in the diseased brain. For example, patients with MCI, and AD compared to healthy controls had significantly higher occurrence, coverage, and duration of microstate A (Musaeus et al., 2019, 2020). In comparison, 40 Hz light flicker significantly reduced the coverage of class A, but did not change the class A duration and occurrence. Studies of patients with schizophrenia showed increased presence of microstate C and decreased presence of microstate D compared to controls (da Cruz et al., 2020), while 40 Hz light flicker significantly increased the occurrence of microstate D compared with the rest state and random flicker (Figure 3D). In the stroke brain, all four microstates are stably expressed (Zappasodi et al., 2017). In patients with a right lesion inducing neglect symptoms, microstate D topography was different. A preserved microstate B in the acute phase of stroke was correlated with a better effective recovery. Together, these association studies warrant further studies to establish the therapeutic impact of 40 Hz light flicker.

Microstate transitioning was slower and less complex in AD. Microstates C and D transitioned significantly more to microstate A in patients with AD compared to healthy controls, and microstate C transitioned more to microstate A in MCI compared to healthy controls (Musaeus et al., 2019, 2020). In comparison, both 40 Hz and random flicker significantly increased C and D transitions (Figure 4H). Because microstate transitioning is non-Markovian, the Lempel–Ziv complexity (LZC) of the microstate transitioning sequence has been developed as a novel measure of microstate complexity (Tait et al., 2020). LZC measurement characterizes the development of spatiotemporal activity patterns in high-dimensionality nonlinear systems, like the brain and heart. The increment in microstate complexity thus indicates a potential augmentation in cognitive functions. For example, a decreased LZC was observed during the progression of MCI and AD (Tait et al., 2020). However, the biological meaning of LZC may not be exactly the same as those indicated by microstate duration, occurrence, and transitioning probabilities. For example, in a study of thalamic ischemic stroke, patients had increased mean LZC than the controls (Liu et al., 2016). Interestingly, the current study demonstrated increased microstate LZCs in response to both 40 Hz and random light flicker compared with the rest state (Figure 5), suggesting that visual light stimulation impacts the LZC. However, it is premature to assume that random flicker would have the same biological effect to the brain microstate as the 40 Hz flicker just because it produced similar LZC.

The current study has limitations. The conclusive link between the current study with the existing literature on microstate alterations in MCI/AD patients is challenging to establish. One striking difference of the present study from published studies was that our test subjects were all young adults in their 20th, limiting its relevance to studies using older subjects. There are also inconsistencies in the literature concerning the alterations of microstates in AD (Dierks et al., 1997; Musaeus et al., 2019, 2020; Smailovic et al., 2019; Smailovic and Jelic, 2019). Therefore, data from a much larger cohort of populations are needed. Importantly, it has been argued that EEG microstate topography is predominantly determined by intracortical sources in the alpha band (Milz et al., 2017). Our data, therefore, focused on the 8–13 Hz alpha band. Thus, it is necessary to compare EEG microstates generated using similar bandwidths in the literature, which is scarce. A dynamic source estimation is a powerful tool yielding millisecond-level cortical neuroactivities. Averaging epoch data potentially covered up numerous temporal information. Collectively, important questions remain whether 40 Hz light flickers can reverse diseased brain microstates and what is the underlying mechanisms associated with the brain response to rhythmic light flickers.

## Data Availability Statement

The datasets presented in this study can be found in online repositories. The names of the repository/repositories and accession number(s) can be found below: Python and R analysis code is available at https://github.com/zhenyumi/LangouEEG.

## Ethics Statement

The studies involving human participants were reviewed and approved by local ethics committees, i.e., the Southern University of Science and Technology Institutional Review Board (SUSTech IRB 2021087). The patients/participants provided their written informed consent to participate in this study.

## Author Contributions

YZ supervised the EEG recordings, designed light device, analyzed data, shared the coding work (GitHub repository), and drafted the manuscript. ZZ participated in EEG recordings, collected and analyzed data, wrote the code (GitHub repository), and drafted the manuscript. LL participated in the EEG recordings, data collection, and analysis. HT analyzed the data and revised the draft manuscript. FC did equipment setup and adjustment, data curation and analysis. S-TH conceived the idea, supervised the project, provided financial support, analyzed the data, and wrote the final draft of the manuscript. All authors contributed to the article and approved the submitted version.

## Conflict of Interest

The authors declare that the research was conducted in the absence of any commercial or financial relationships that could be construed as a potential conflict of interest.

## Publisher’s Note

All claims expressed in this article are solely those of the authors and do not necessarily represent those of their affiliated organizations, or those of the publisher, the editors and the reviewers. Any product that may be evaluated in this article, or claim that may be made by its manufacturer, is not guaranteed or endorsed by the publisher.
