# Supplementary material for: 40 Hz Light Flicker Alters Human Brain Electroencephalography Microstates and Complexity Implicated in Brain Diseases
Source: Front Neurosci. 2021 Dec 13;15:777183. doi: 10.3389/fnins.2021.777183 (PMC8710722; doi:10.3389/fnins.2021.777183)

Supplementary Material

40 Hz Light Flicker Alters Human Brain Electroencephalography Microstates and Complexity Implicated in Brain Diseases

Yiqi Zhang[1#], Zhenyu Zhang[1#], Lei Luo[1], Huaiyu Tong[2], Fei Chen[3], Sheng-Tao Hou[1*]

[1] Brain Research Centre and Department of Biology, Southern University of Science and Technology, Shenzhen, China,

[2] Department of Neurosurgery, The First Medical Center, Chinese PLA General Hospital, Beijing, China,

[3] Department of Electrical and Electronic Engineering, Southern University of Science and Technology, Shenzhen, China

Supplementary Figure 1 | Percent power changes between 40 Hz and total power in position O of all test subjects. (**A**) is relative to random light flicker, while (**B**) is relative to 40 Hz light stimulation.

Figure S1

A Relative Power Change at Position O: Response to Random Flicker

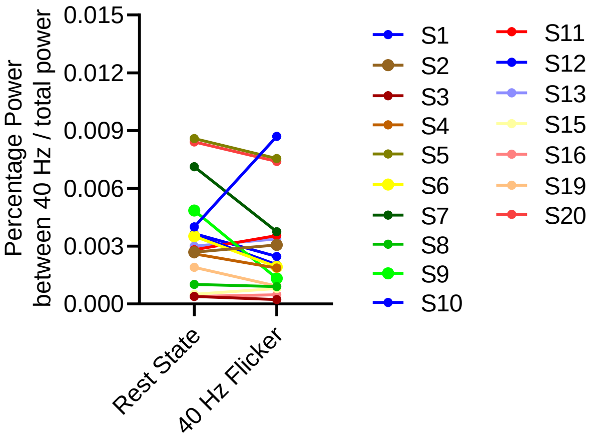

B Relative Power Change at Position O: Response to 40 Hz Flicker

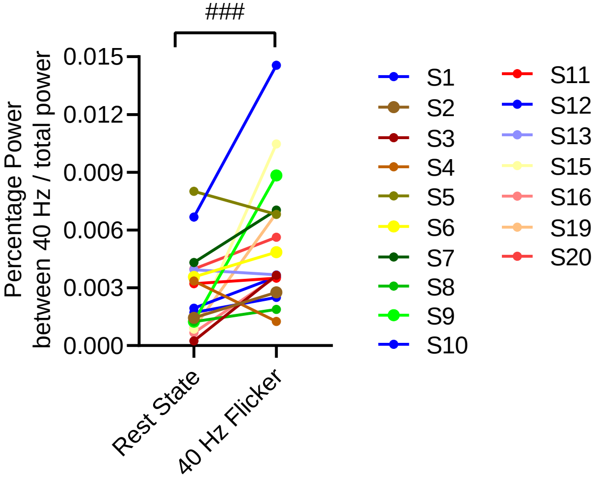

Supplement: Supplementary file 1 [file Data_Sheet_1.pdf]
